# Seasonal trends in nesting leatherback turtle (*Dermochelys coriacea*) serum proteins further verify capital breeding hypothesis

**DOI:** 10.1093/conphys/cou002

**Published:** 2014-02-18

**Authors:** Justin R. Perrault, Jeanette Wyneken, Annie Page-Karjian, Anita Merrill, Debra L. Miller

**Affiliations:** 1 Department of Biological Sciences, Florida Atlantic University, 136 Sanson Science, 777 Glades Road, Boca Raton, FL 33431, USA; 2 The University of Georgia, College of Veterinary Medicine, 501 D.W. Brooks Drive, Athens, GA 30602, USA; 3 The University of Georgia, College of Veterinary Medicine, Veterinary Diagnostic and Investigational Laboratory, 43 Brighton Road, Tifton, GA 31793, USA

**Keywords:** Capital breeding, *Dermochelys coriacea*, leatherback sea turtle, nesting, protein electrophoresis, serum

## Abstract

Serum protein concentrations provide insight into the nutritional and immune status of organisms. It has been suggested that some marine turtles are capital breeders that fast during the nesting season. In this study, we documented serum proteins in neophyte and remigrant nesting leatherback sea turtles (*Dermochelys coriacea*). This allowed us to establish trends across the nesting season to determine whether these physiological parameters indicate if leatherbacks forage or fast while on nesting grounds. Using the biuret method and agarose gel electrophoresis, total serum protein (median = 5.0 g/dl) and protein fractions were quantified and include pre-albumin (median = 0.0 g/dl), albumin (median = 1.81 g/dl), α_1_-globulin (median = 0.90 g/dl), α_2_-globulin (median = 0.74 g/dl), total α-globulin (median = 1.64 g/dl), β-globulin (median = 0.56 g/dl), γ-globulin (median = 0.81 g/dl) and total globulin (median = 3.12 g/dl). The albumin:globulin ratio (median = 0.59) was also calculated. Confidence intervals (90%) were used to establish reference intervals. Total protein, albumin and total globulin concentrations declined in successive nesting events. Protein fractions declined at less significant rates or remained relatively constant during the nesting season. Here, we show that leatherbacks are most likely fasting during the nesting season. A minimal threshold of total serum protein concentrations of around 3.5–4.5 g/dl may physiologically signal the end of the season's nesting for individual leatherbacks. The results presented here lend further insight into the interaction between reproduction, fasting and energy reserves and will potentially improve the conservation and management of this imperiled species.

## Abstract (precis)

In nesting leatherback turtles from St. Croix, we found that total protein, albumin and total globulin concentrations declined in successive nesting events, while protein fractions (alpha-, beta- and gamma-globulins) declined at less significant rates or remained relatively constant. These results suggest that leatherbacks are fasting during the nesting season.

## Introduction

Parental care in reptiles rarely extends further than nest site selection and the nutrients that are provisioned within the albumen and yolk of the egg (Miller, 1997; Hughes and Hughes, 2006; Patel, 2013). While parental care is minimal in this vertebrate class, these organisms still incur costs to body condition and possibly health in order to reproduce successfully (i.e., maternal investment; Trivers, 1972; Harshman and Zera, 2007). Reptiles, including marine turtles, undergo energy trade-offs between the two extremely costly life functions of maintaining normal physiological processes and reproduction (Le Maho, 2002; Plot *et al.*, 2013). Given that reproduction is costly, organisms must use one of the following two mechanisms to compensate for the resources lost during this process: (i) income breeding, when an organism forages while reproducing; or (ii) capital breeding, when an organism maintains energy for reproductive processes through stored resources (Stephens *et al.*, 2009; Plot *et al.*, 2013).

Marine turtles are thought to use a capital breeding strategy, whereby large amounts of prey are ingested on foraging grounds and stored as fat reserves (Hamann *et al.*, 2003; Goldberg *et al.*, 2013). In some cases, opportunistic feeding by marine turtles while on breeding and nesting grounds has been proposed, but is likely to be negligible (Schoefield *et al.*, 2006; Casey *et al.*, 2010). Sea turtles are unique among reptiles in that they embark on extremely long-distance migrations (>13 000 km in the Atlantic; Eckert *et al.*, 2012, for review) from foraging grounds to nesting and breeding grounds (Plotkin, 2003); these journeys are powered by fat stores that accumulate on foraging grounds (Wallace *et al.*, 2006). In particular, leatherback turtles make the most extensive migrations (Eckert *et al.*, 2012, for review), which are fuelled by gelatinous prey items that have relatively dilute energetic value (Bjorndal, 1997; Houghton *et al.*, 2006; Doyle *et al.*, 2007). On foraging grounds, western Atlantic leatherbacks are known to ingest over 300 kg of food/day (up to 840 kg/day; Heaslip *et al.*, 2012). Although the energy density of their prey items is low, the massive amount of food ingested on foraging grounds is sufficient to fuel migrations southward and provide enough energy to sustain the largest reproductive output of all marine turtles (37–62 kg of eggs per season laid across six to 10 clutches; Miller, 1997; Wallace *et al.*, 2006; Guirlet *et al.*, 2008; Plot *et al.*, 2013).

A substantial decrease in food intake during the nesting season is expected to influence biochemical parameters (Plot *et al.*, 2013), including serum proteins and their fractions. Serum proteins provide insight into the nutritional status of an organism, allowing for evaluation of the hydration and immune status, as well as identification of the presence of inflammation or infectious disease (Campbell, 2006; Evans, 2011). Several studies report protein fractions in nesting marine turtles (Deem *et al.*, 2006, 2009; Perrault *et al.*, 2012), and one study (Honarvar *et al.*, 2011) describes changes in proteins [total protein (TP), albumin and globulin] across the nesting season. The measures of Honarvar *et al.* (2011) are based upon a hand-held refractometer and a field chemistry analyser. These methods are considered less accurate than the laboratory-based electrophoretic measures used in other studies (Cray and Tatum, 1998; Müller and Brunnberg, 2010).

In very few studies of marine turtles, changes in biochemical parameters across the nesting season have been measured (Hamann *et al.*, 2002; Honarvar *et al.*, 2011; Perrault *et al.*, 2012; Goldberg *et al.*, 2013; Plot *et al.*, 2013). Here we established the largest sample size of serum proteins and their fractions in leatherback sea turtles to date, describe seasonal changes of these physiological parameters in leatherbacks nesting on Sandy Point National Wildlife Refuge (SPNWR), St Croix, US Virgin Islands, and verify that leatherbacks are capital breeders that fast during the nesting season.

## Materials and methods

### Ethical procedures

Sampling on SPNWR was conducted in conjunction with the West Indies Marine Animal Research and Conservation Service (WIMARCS) saturation-tagging project and was carried out in accordance with a National Marine Fisheries Service Special Use Permit # 41526-2009-004. Florida Atlantic University's Institutional Animal Care and Use Committee approved this study (protocol #A07-03).

### Study period and nesting beach

**Figure 1:**
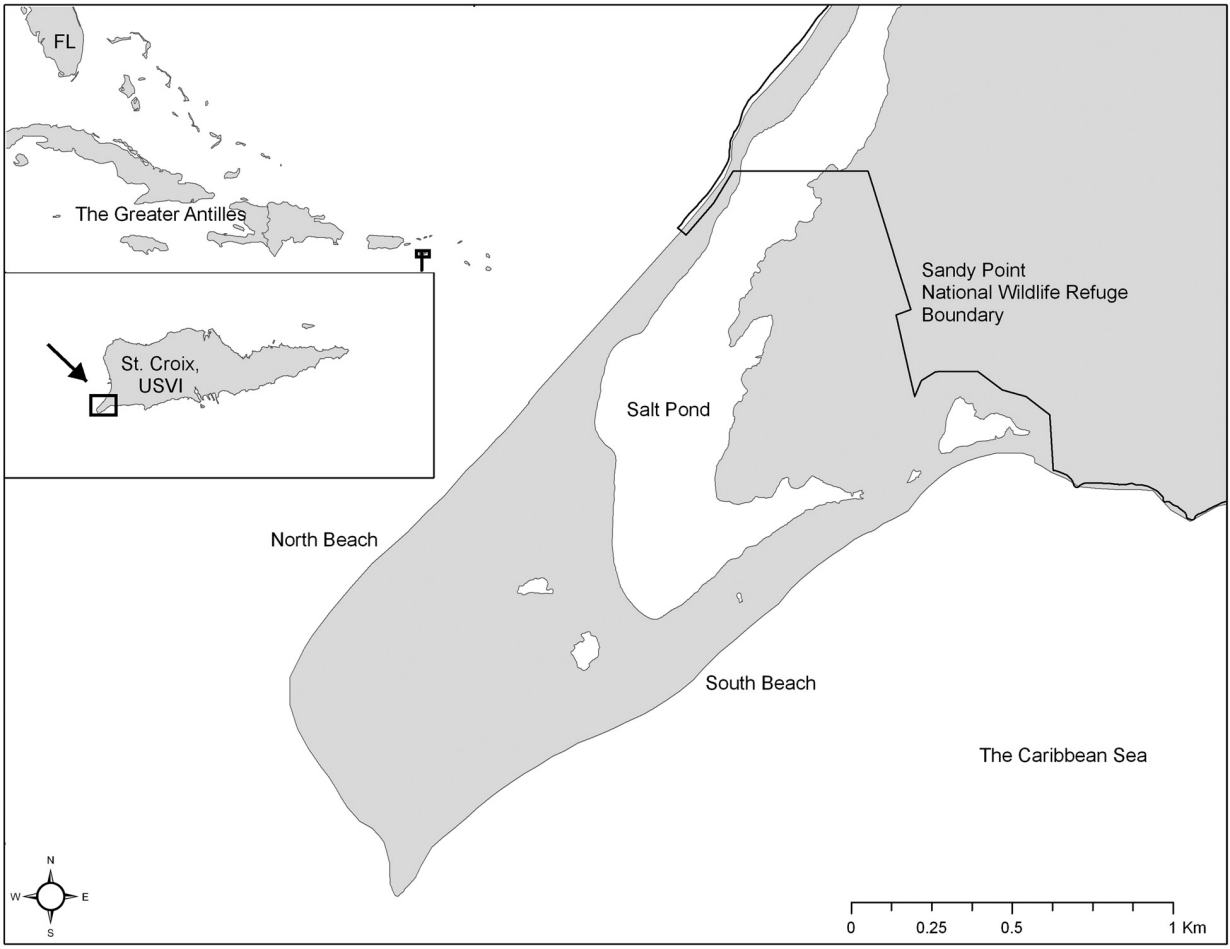
Sandy Point National Wildlife Refuge, St Croix, US Virgin Islands, nesting beach.

Leatherback sea turtles were sampled along the beach (2.4 km) at SPNWR (17°40′40′′ N, 64°54′0′′ W; Fig. 1) during their nesting processes. We sampled individuals from 1 April to 15 July 2009. Leatherbacks typically nest six to eight times (or more) in a season (Miller, 1997). Nesting females on SPNWR exhibit high nest site fidelity, which allowed us to repeatedly sample individuals multiple times within the season. Over 1000 individual leatherbacks have been tagged at SPNWR since tagging programs began in 1979 (Garner and Garner, 2010).

### Animals and samples

The beach was patrolled on foot nightly for nesting activity from 20.00 to 05.00 h, or until the last female finished nesting. Turtles were identified by passive integrative transponder (PIT) tags and/or flipper tags. These tags were applied to nesting females that lacked either of these tag types. Tagging and blood collection from the hindlimb rete system of nesting leatherback turtles occurred during their nesting fixed action pattern (Dutton and Dutton, 1994; Dutton, 1996; Perrault *et al.*, 2012). During oviposition, the sampling site was swabbed with betadine, and ∼7 ml of blood was collected aseptically into 10 ml serum separator (red-top) Vacutainer^®^ tubes using 18 gauge venous collection needles fitted into Vacutainer^®^ tube holders. After blood collection, the area was cleansed with betadine, and pressure was applied to promote haemostasis. Blood samples were chilled immediately on ice. Serum separator tubes were spun within 12 h of collection using a centrifuge at ∼1400 *g* for 5 min. The serum was collected and separated into 2 ml Corning^®^ cryogenic vials. When possible, subsequent samples were collected from the same nesting females during successive nesting events (8–12 days later). Serum was frozen at −20°C for 1 month before shipping on ice to the Veterinary Diagnostic and Investigational Laboratory at the University of Georgia (UGA-VDIL, Tifton, GA, USA) for analyses.

### Protein electrophoresis

Haemolysed samples or those exhibiting signs of lipaemia were removed from analyses. Total serum protein (TP) was determined by the biuret method (Siemens ADVIA 1200 Clinical Chemistry System, Siemens Medical Solutions Diagnostics, Tarrytown, NY, USA). Albumin, α-, β- and γ-globulins were determined by agarose gel electrophoresis using a Helena SPIFE 3000 system (Split Beta SPE, Helena Laboratories, Beaumont, TX, USA). Gels were stained with an acid blue stain and then scanned on a QuickScan 2000 densitometer (Helena Laboratories). Protein fractions were visually identified on the electrophoretogram and calculated by densitometry (percentage area under the curve multiplied by total serum protein concentration). Running conditions were 650 V for 6 min. Dual analyses were run for each sample and then averaged. The albumin:globulin (A:G) ratio was calculated. Quality control was carried out using serum protein electrophoresis (SPE) normal and SPE abnormal (Helena Laboratories) controls on each gel run.

### Statistical analyses

A general linear model was used to compare TP concentrations in remigrant nesters and neophytes (new nesters). The date of the nesting event was treated as a random factor. Repeated-measures ANOVAs were carried out to determine whether TP concentrations, protein fractions and/or the A:G ratio significantly differed in subsequent nesting events. We were able to assign the nesting females' nest number accurately based on the date observed at first nesting and knowing the nesting fidelity of turtles to SPNWR. For example, if a female's first observed nesting event was 1 April and she was then observed nesting again on 20 April, we predicted that 20 April was her third nesting event (internesting interval is 10 days on average). This allowed us to remove time as a covariate from the analyses. Additionally, TP and protein fraction concentrations from each predicted nest number (one to eight) were averaged, and regression analyses were appropriately fitted to the trends (linear, logarithmic or polynomial regressions). Sample sizes for nest numbers nine and 10 were low and were not used in statistical analyses, but are provided in the figures for comparison. In a few cases, nest numbers could not be estimated and, consequently, we did not include these samples in the analyses. Lastly, linear regression lines of TP, albumin and globulin were compared using Student's *t* test for slope of a regression line (Zar, 1999). Data were analysed using SPSS, version 21.0 (SPSS, Inc., Chicago, IL, USA).

## Results

**Figure 2:**
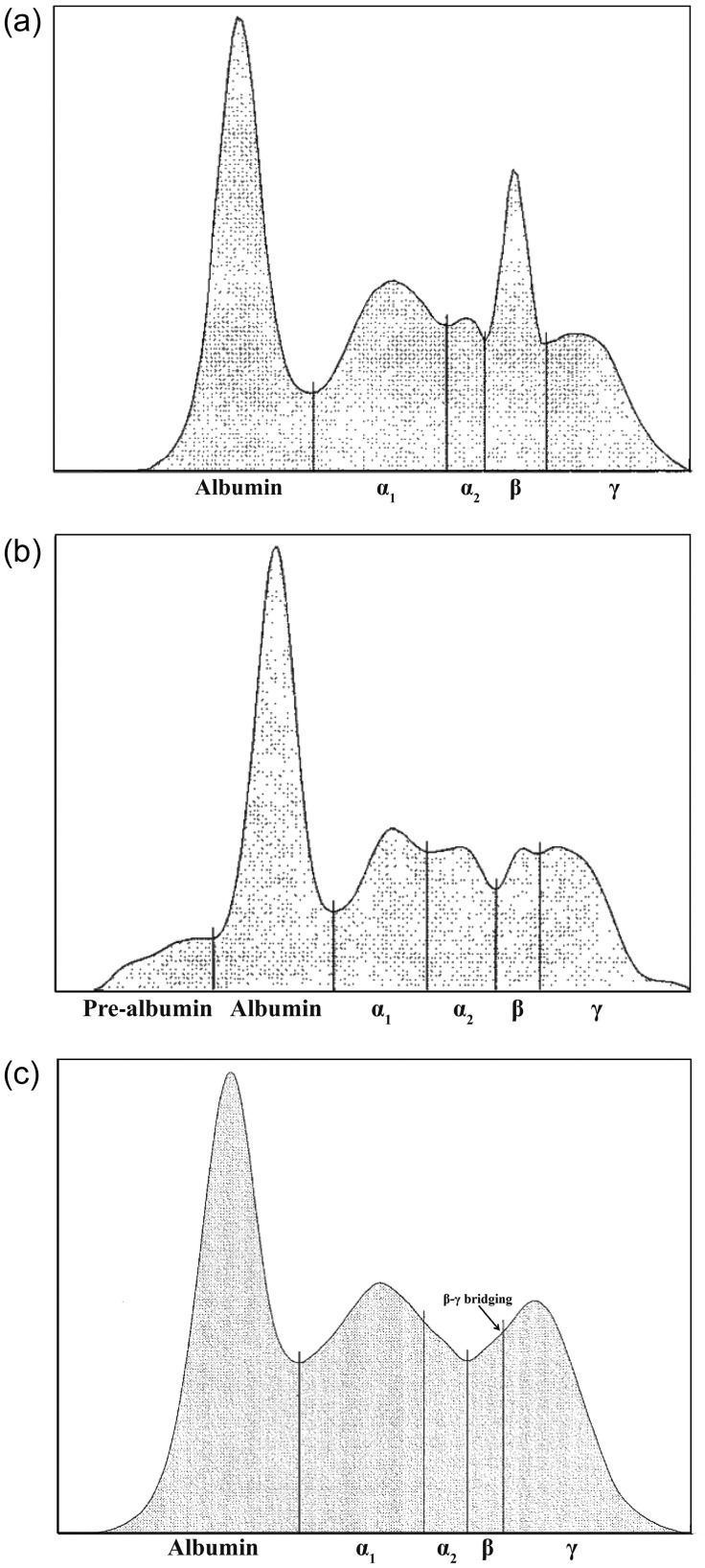
(**a**) Serum protein electrophoretogram of a nesting leatherback sea turtle. Albumin, α-, β- and γ-globulin fractions are visible. (**b**) Serum protein electrophoretogram of a leatherback sea turtle with a pre-albumin band. (**c**) Serum protein electrophoretogram of a leatherback sea turtle with β-γ bridging.

A total of 217 samples (from 76 turtles, both neophytes and remigrants) were collected during the 2009 nesting season on SPNWR (see Perrault *et al.*, 2013 for morphometric data of nesting females; the minimum curved carapace length ranged from 138 to 174 cm). Protein fractions (pre-albumin, albumin, α-, β- and γ-globulins; Fig. 2) were determined for 129 of the samples (41 remigrants only; Table 1).

**Table 1:** Synopsis of plasma protein electrophoretic values for nesting leatherback sea turtles on Sandy Point National Wildlife Refuge, St Croix, US Virgin Islands

Parameters	Outliers included				Outliers excluded			
	*n*	Mean ± SD and/or median	Range	Reference interval	*n*	Mean ± SD and/or median	Range	Reference interval
Total protein (g/dl)	217	4.96 ± 0.73; 5.00	3.20–6.90	3.79–6.31	217	4.96 ± 0.73; 5.00	3.20–6.90	3.79–6.31
Pre-albumin (g/dl)	129	0.00	0.00–0.13	0.00–0.04	121	0.00 ± 0.00; 0.00	0.00–0.00	0.00–0.00
Albumin (g/dl)	129	1.81	1.20–3.01	1.45–2.32	126	1.81 ± 0.25; 1.81	1.20–2.48	1.44–2.27
α_1_-Globulin (g/dl)	129	0.90	0.46–2.24	0.54–1.63	125	0.89	0.46–1.66	0.54–1.39
α_2_-Globulin (g/dl)	129	0.74	0.13–2.03	0.22–1.45	128	0.74	0.13–1.68	0.22–1.44
Total α-Globulin (g/dl)	129	1.69 ± 0.42; 1.64	0.90–2.91	1.03–2.56	126	1.66 ± 0.38; 1.64	0.90–2.65	1.03–2.33
β-Globulin (g/dl)	129	0.56	0.20–1.53	0.26–1.06	124	0.55	0.20–1.06	0.25–0.97
γ-Globulin (g/dl)	129	0.81 ± 0.19; 0.81	0.33–1.47	0.50–1.11	123	0.80 ± 0.16; 0.80	0.42–1.17	0.52–1.06
Globulin (g/dl)	129	3.06 ± 0.50; 3.12	2.02–4.36	2.26–3.91	129	3.06 ± 0.50; 3.12	2.02–4.36	2.26–3.91
Albumin:globulin ratio	129	0.60 ± 0.09; 0.59	0.41–0.81	0.47–0.76	129	0.60 ± 0.09; 0.59	0.41–0.81	0.47–0.76

Statistics for plasma proteins are given with and without outliers (<or>1.5 interquartile range from the minimum or maximum values). Reference intervals are given as 90% confidence limits (central 90% of the values, 5th–95th percentiles).

Using agarose gel electrophoresis, five or six protein fractions were characterized in nesting female leatherbacks. These included albumin, α-globulin (separated into α_1_- and α_2_-globulins), β-globulin and γ-globulin fractions (Fig. 2a). A pre-albumin band was observed in eight of the samples (Fig. 2b). Figure 2c shows the presence of β-γ bridging, which was present in six samples (∼4%). Reference intervals for nesting female leatherbacks were determined using 90% confidence intervals (non-parametric method; ASVCP, 2014) and are shown in Table 1. Comparisons of TP and protein fractions with those of other marine turtles from the literature are given in Table 2 (methodologies of each study are given as footnotes within the table). Total protein concentrations in neophytes and remigrants did not differ significantly (*P* > 0.05) and were treated as one group.

**Table 2:** Concentrations of plasma proteins and protein fractions (in grams per decilitre) of marine turtles in this study and the literature

Location	Ocean	Sex	*n*	Study	Total [protein]	[Albumin]	[α_1_-Globulin]	[α_2_-Globulin]	Total [α-Globulin]	[β-Globulin]	[γ-Globulin]	Globulin	A:G
Leatherbacks													
Gabon, Africa	East Atlantic	NF	8–12	1^a^	4.0 ± 1.0^b^ (3.0–5.0)^b^	1.2 ± 0.4 (1.1–2.4)	0.2 ± 0.1 (0.1–0.3)	0.8 ± 0.2 (0.5–1.2)	–	0.8 ± 0.1 (0.6–0.9)	0.8 ± 0.2 (0.5–1.3)	–	∼0.46
Trinidad	Northwest Atlantic	NF	13	2^a^	4.9 ± 0.6 (4.1–6.2)	–	–	–	–	–	–	–	–
East Coast USA	Northwest Atlantic	FM, FF	18	3^a^	–	1.8 ± 0.4 (0.9–2.4)	0.5 ± 0.2 (0.1–0.8)	0.4 ± 0.3 (0–0.9)	–	0.8 ± 0.0.2 (0.4–1.0)	–	–	–
East Coast USA	Northwest Atlantic	FM	9	3^a^	5.0 ± 0.7^c^ (4.3–6.2)^c^	–	–	–	–	–	1.5 ± 0.4^c^ (0.8–1.9)^c^	3.2 ± 0.4^c^ (2.6–4.0)^c^	∼0.55
East Coast USA	Northwest Atlantic	FF	4	3^a^	5.2 ± 0.6^c^ (4.4–5.9)^c^	–	–	–	–	–	1.6 ± 0.2^c^ (1.4–1.9)^c^	3.3 ± 0.4^c^ (2.9–3.9)^c^	∼0.56
CA, USA	East Pacific	FM	5	4^a^	5.3 (3.8–5.9)	2.1 (1.2–2.4)	–	–	–	–	–	3.1 (2.6–3.8)	∼0.67
CA, USA	East Pacific	FF	9	4^a^	4.9 (3.7–5.9)	1.9 (1.4–2.7)	–	–	–	–	–	3.1 (2.1–3.6)	∼0.61
Costa Rica	East Pacific	NF	8	4^a^	3.9 (3.6–4.4)	1.7 (1.4–2.0)	–	–	–	–	–	2.4 (2.0–2.5)	∼0.71
Papua New Guinea	West Pacific	NF	11	4^a^	4.2 (3.5–5.2)	1.9 (1.5–2.3)	–	–	–	–	–	2.3 (2.0–2.9)	∼0.83
St Croix, US Virgin Islands	Northwest Atlantic	NF	12	4^a^	4.1 (2.8–4.7)	1.7 (1.2–2.0)	–	–	–	–	–	2.3 (1.6–2.7)	∼0.74
Equatorial Guinea	East Atlantic	NF	54–55	5^a^	5.1 ± 0.1 (3.6–6.6)^d^	1.8 ± 0.03 (1.3–2.2)^d^	–	–	–	–	–	2.4 ± 0.04 (1.8–3.0)^d^	∼0.75
FL, USA	Northwest Atlantic	NF	66	6^a^	3.9 ± 0.6 (2.0–5.1)	1.5 ± 0.3 (0.7–2.2)	–	–	0.9 (0.3–2.1)	0.7 (0.2–1.9)	0.7 ± 0.2 (0.2–1.3)	2.3 ± 0.4 (1.2–3.7)	0.70 (0.30–1.70)
St Croix, US Virgin Islands	Northwest Atlantic	NF	129–217	Present study^a^	5.0 ± 0.7 (3.2–6.9)	1.8 (1.2–3.0)	0.9 (0.5–2.2)	0.74 (0.1–2.0)	1.7 ± 0.4 (0.9–2.9)	0.6 (0.2–1.5)	0.8 ± 0.2 (0.3–1.5)	3.1 ± 0.5 (2.0–4.4)	0.60 ± 0.09 (0.41–0.81)
Loggerheads									0.5 ± 0.1 (0.3–0.7)	0.8 ± 0.1 (0.6–1.1)	1.8 ± 0.6 (1.3–2.9)	–	0.33 ± 0.10 (0.17–0.45)
Florida Bay, USA	Northwest Atlantic	AF	7	7^a^	4.4 ± 0.8 (3.3–5.4)	1.0 ± 0.1 (0.9–1.3)	–	–	0.5 ± 0.1 (0.4–0.6)	0.8 ± 0.1 (0.6–1.0)	2.1 ± 0.6 (1.1–3.0)	–	0.30 ± 0.62 (0.18–0.38)
Florida Bay, USA	Northwest Atlantic	AM	7	7^a^	4.6 ± 0.3 (4.2–5.2)	1.1 ± 0.1 (0.9–1.3)	–	–	0.5 ± 0.1 (0.4–0.8)	1.0 ± 0.2 (0.7–1.3)	2.0 ± 0.3 (1.6–2.5)	–	0.32 ± 0.05 (0.23–NA)
Florida Bay, USA	Northwest Atlantic	JF	7	7^a^	3.9 ± 0.8 (2.9–5.2)	1.0 ± 0.2 (0.8–1.3)	–	–	0.4 ± 0.1 (0.4–0.5)	0.6 ± 0.1 (0.5–0.7)	1.9 ± 0.8 (0.8–2.9)	–	0.38 ± 0.15 (0.25–0.63)
FL and GA, USA	Northwest Atlantic	FF, FM	39	8^a^	3.7 ± 1.1 (1.6–5.6)	0.8 ± 0.3 (0.3–1.2)	0.1 ± 0.1 (0.1–0.3)	0.1 (0.1–0.3)	–	1.0 ± 0.3 (0.5–1.5)	1.6 ± 0.7 (0.5–2.9)	2.9 ± 0.9 (1.0–4.0)	∼0.44
GA, USA	Northwest Atlantic	NF	24–25	8^a^	5.2 ± 0.5 (4.6–6.1)	1.2 ± 0.3 (1.0–1.8)	0.2 ± 0.1 (0.1–0.3)	0.2 (0.1–1.0)	–	1.7 ± 0.5 (0.9–2.6)	1.2 ± 0.4 (0.7–2.2)	4.0 ± 0.7 (2.7–5.1)	∼0.43
GA, USA	Northwest Atlantic	SF, SM	8–13	8^a^	2.5 ± 0.8 (0.4–3.9)	0.6 ± 0.4 (0.2–1.2)	0.1 ± 0.03 (0.03–0.1)	0.2 (0.1–0.3)	–	0.7 ± 0.5 (0.2–1.6)	1.0 ± 0.4 (0.5–1.6)	1.7 ± 0.7 (0.1–2.4)	∼0.41
FL, USA	Northwest Atlantic	All	437	9^a^	3.3 (1.4–6.3)	1.0 (0.2–2.0)	–	–	0.5 (0.1–1.4)	0.8 (0.3–1.8)	1.0 (0.3–3.3)	–	0.43 (NA–0.89)
Green turtles													
FL, USA	Northwest Atlantic	JF, JM	152	9^a^	3.6 (1.7–6.2)	1.5 (0.7–2.3)	–	–	0.5 (0.3–0.9)	0.7 (0.2–1.3)	0.9 (0.2–2.3)	–	0.69 (0.42–1.19)

Concentrations are given as the mean ± SD or median (range). Abbreviations: AF, adult female; AM, adult male; FF, foraging female; FM, foraging male; JF, juvenile female; JM, juvenile male; NA, not available; NF, nesting female; SF, stranded female; SM, stranded male; and TP, total protein. The studies are numbered as follows: 1, Deem *et al.* (2006); 2, Harms *et al.*, 2007; 3, Innis *et al.* (2010); 4, Harris *et al.* (2011); 5, Honarvar *et al.* (2011); 6, Perrault *et al.* (2012); 7, Gicking *et al.* (2004); 8, Deem *et al.* (2009); and 9, Osborne *et al.* (2010).

^a^1 = TP by clinical chemistry analyser, fractions by electrophoresis; 2 = clinical chemistry analyser; 3 = TP by clinical chemistry analyser, fractions by electrophoresis; 4 = clinical chemistry analyser; 5 = TP by handheld refractometer, fractions by clinical chemistry analyser; 6 = TP by biuret method, fractions by electrophoresis; present study = TP by biuret method, fractions by electrophoresis; 7 = TP by biuret method, fractions by electrophoresis; 8 = TP and globulin by clinical chemistry analyser, fractions by electrophoresis; and 9 = TP by biuret method, fractions by electrophoresis.

^b^Reported TP concentrations in blood collected from lithium heparin and sodium heparin tubes. Lithium heparin values are shown in the table, although the values were not statistically different. Sodium heparin values = 4.6 ± 1.0 g/dl (3.2–6.0 g/dl).

^c^Values that were statistically different between sexes were reported separately.

^d^Values reported are reference values, not the true range.

**Figure 3:**
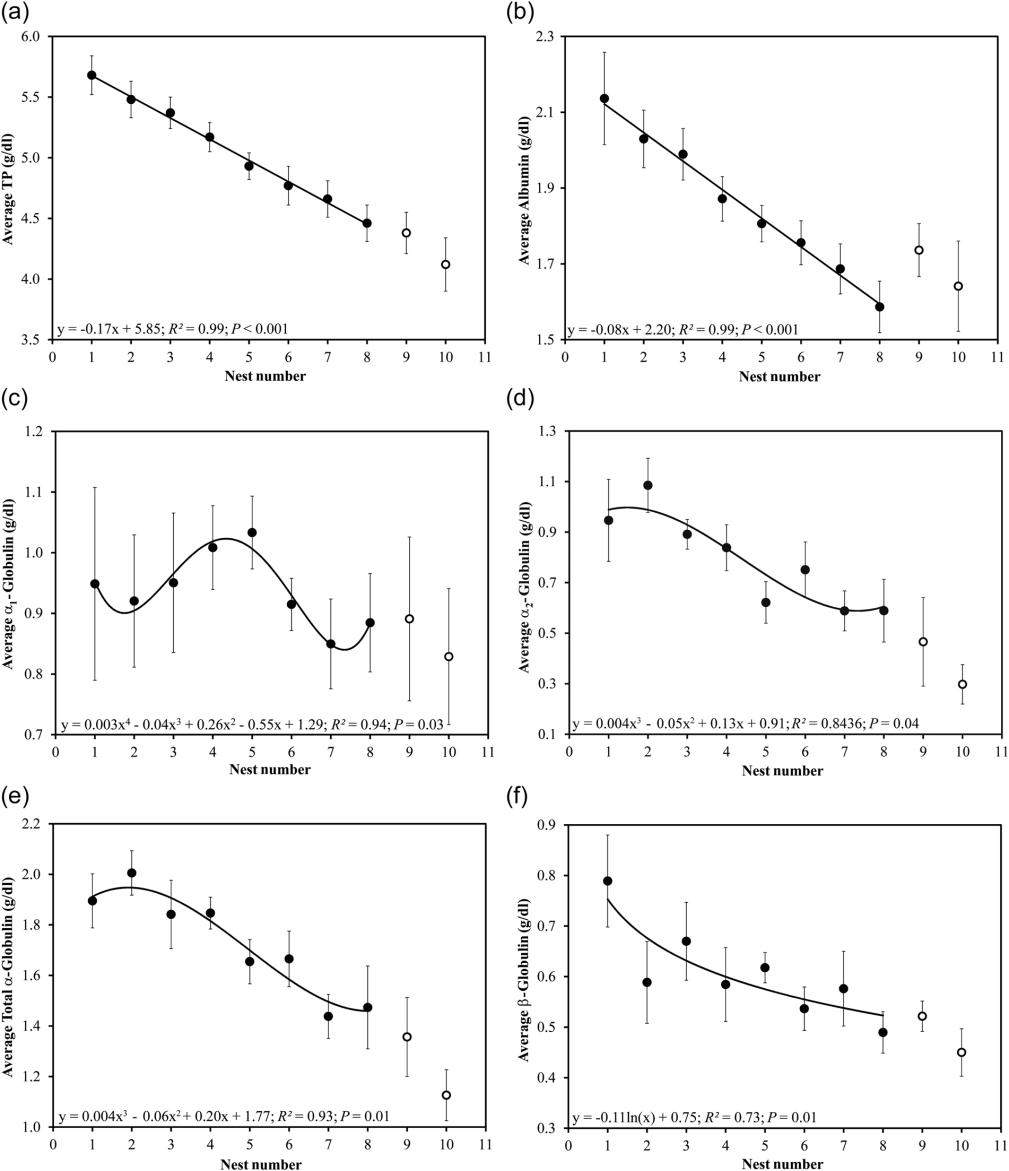

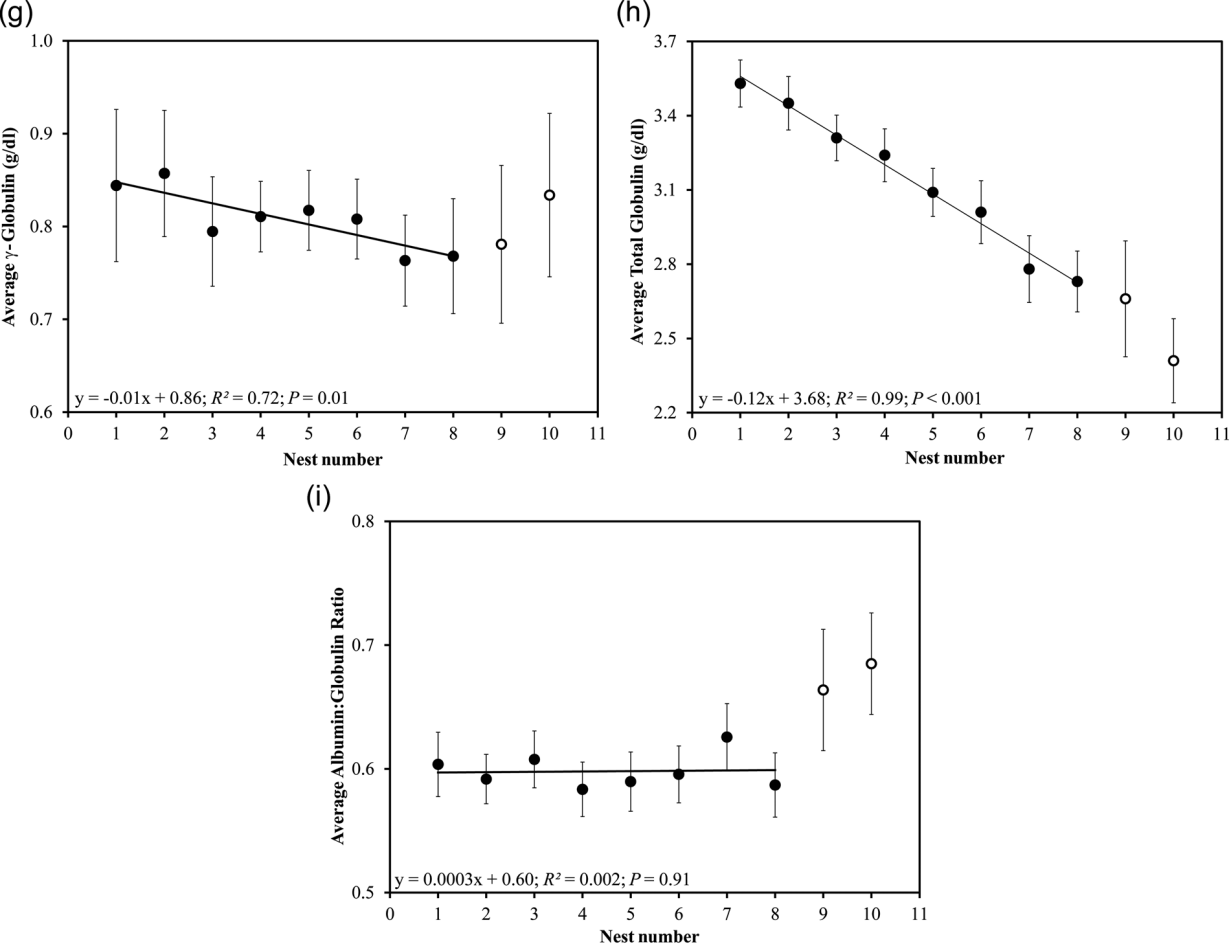
Trends in nesting leatherback total protein (TP; a), albumin (b), α_1_-globulin (c), α_2_-globulin (d), total α-globulin (e), β-globulin (f), γ-globulin (g), total globulin (h) and the albumin:globulin (A:G) ratio (i). Values are shown as means ± SEM for each nest number. The means ± SEM from the ninth and 10th nesting events are shown in the figures (open circles); however, these values were not included in statistical analyses owing to low sample sizes (*n* ≤ 6).

**Figure 4:**
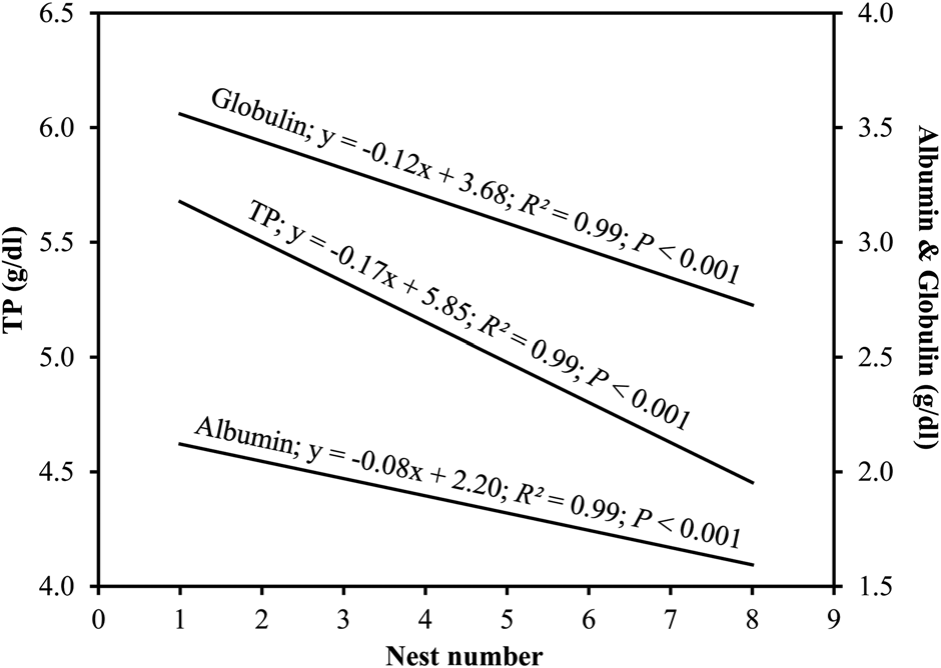
Linear regression lines of TP, albumin and globulin during each nesting event. The slopes for TP and albumin and for albumin and globulin were significantly different. The slopes for TP and globulin did not differ significantly.

Total protein concentrations declined significantly in each successive nesting event (least-squares linear regression; *r*^2^ = 0.99, *P* < 0.001; Fig. 3a). Albumin declined linearly in each successive nesting event (*r*^2^ = 0.99, *P* < 0.001; Fig. 3b). Polynomial regressions indicated that the α-globulins fluctuated (α_1_, *r*^2^ = 0.94, *P* = 0.03; Fig. 3c) or gradually declined (α_2_, *r*^2^ = 0.84, *P* = 0.04; Fig. 3d; and total α, *r*^2^ = 0.93, *P* = 0.01, Fig. 3e) during the nesting season. The β-globulins decreased logarithmically (*r*^2^ = 0.73, *P* = 0.01; Fig. 3f), while the γ-globulin fraction decreased in a stepwise manner during the nesting season (*r*^2^ = 0.72, *P* = 0.01; Fig. 3g). Globulin declined linearly in each successive nesting event (*r*^2^ = 0.99, *P* < 0.001; Fig. 3h). Lastly, the A:G ratio remained constant during subsequent nesting events (*r*^2^ = 0.002, *P* = 0.91; Fig. 3i); however, albumin decreased at a significantly lesser rate across the nesting season when compared with TP (*t*_1,12_ = 15.55, *P* < 0.001; Fig. 4) and globulin (*t*_1,12_ = 2.78, *P* = 0.02; Fig. 4). Total protein and globulin declined at similar rates (*P* > 0.05; Fig. 4).

The results of repeated-measures ANOVAs indicated significant declines across the nesting season in TP, albumin and globulin (Tables 3–5; see Supplemental Table 1 for results of repeated-measures ANOVAs). Few significant differences were observed in the individual protein fractions (α_1_-, α_2_-, total α-, β- and γ-globulin and the A:G ratio) across the nesting season (see Supplemental Table 1 for results of repeated-measures ANOVAs), most likely due to small sample sizes.

**Table 3:** Differences in TP concentrations between nesting events (determined by repeated-measures ANOVAs)

	1	2	3	4	5	6	7	8	9	10
1	–	ND	ND	ND	1*	1**	1*	–	–	–
2	ND	–	ND	2**	2*	2**	2**	2***	2**	2**
3	ND	ND	–	3**	3***	3***	3**	3***	3**	–
4	ND	2**	3**	–	ND	ND	4***	4***	4**	4***
5	1*	2*	3***	ND	–	5**	5*	5***	5**	5*
6	1**	2**	3***	ND	5**	–	ND	6**	6**	–
7	1*	2**	3**	4***	5*	ND	–	7**	ND	–
8	–	2***	3***	4***	5***	6**	7**	–	ND	8*
9	–	2**	3**	4**	5**	6**	ND	ND	–	ND
10	–	2**	–	4**	5*	–	–	8*	ND	–

ND represents no significant difference between the sampling events. The number in each cell represents the nesting event with the higher TP concentration. Dashes indicate that sample sizes were too low for comparison.

*Significantly different at *P* ≤ 0.05.

**Significantly different at *P* ≤ 0.01.

***Significantly different at *P* ≤ 0.001.

**Table 4:** Differences in albumin concentrations between nesting events (determined by repeated-measures ANOVAs)

	1	2	3	4	5	6	7	8	9	10
1	–	ND	ND	ND	ND	ND	ND	–	–	–
2	ND	–	ND	2**	2*	2*	2*	2***	–	–
3	ND	ND	–	3*	3***	3**	–	–	–	–
4	ND	2**	3*	–	ND	ND	ND	–	–	–
5	ND	2*	3***	ND	–	5***	5**	5*	–	–
6	ND	2*	3**	ND	5***	–	ND	6*	–	–
7	ND	2*	–	ND	5**	ND	–	ND	–	–
8	–	2***	–	–	5*	6*	ND	–	–	–
9	–	–	–	–	–	–	–	–	–	ND
10	–	–	–	–	–	–	–	–	ND	–

ND represents no significant difference between the sampling events. The number in each cell represents the nesting event with the higher albumin concentration. Dashes indicate that sample sizes were too low for comparison.

*Significantly different at *P* ≤ 0.05.

**Significantly different at *P* ≤ 0.01.

***Significantly different at *P* ≤ 0.001.

**Table 5:** Differences in globulin concentrations between nesting events (determined by repeated-measures ANOVAs)

	1	2	3	4	5	6	7	8	9	10
1	–	ND	ND	ND	1*	–	–	–	–	–
2	ND	–	2*	2*	ND	ND	ND	2**	–	–
3	ND	2*	–	ND	3*	3**	–	–	–	–
4	ND	2*	ND	–	ND	ND	4*	–	–	–
5	1*	ND	3*	ND	–	ND	ND	5*	–	–
6	ND	ND	3**	ND	ND	–	ND	6**	–	–
7	ND	ND	–	4*	ND	ND	–	ND	–	–
8	–	2**	–	–	5*	6**	ND	–	–	–
9	–	–	–	–	–	–	–	–	–	ND
10	–	–	–	–	–	–	–	–	ND	–

ND represents no significant difference between the sampling events. The number in each cell represents the nesting event with the higher globulin concentration. Dashes indicate that sample sizes were too low for comparison.

*Significantly different at *P* ≤ 0.05.

**Significantly different at *P* ≤ 0.01.

## Discussion

### Total protein

#### Population comparisons

Total protein concentrations in nesting females from SPNWR were similar to those of nesting (Trinidad and Equatorial Guinea) and foraging leatherbacks (eastern and western Atlantic Ocean and eastern Pacific; Harms *et al.*, 2007; Innis *et al.*, 2010; Harris *et al.*, 2011; Honarvar *et al.*, 2011). Total protein concentrations in leatherbacks from St Croix were higher than those of nesting leatherbacks from Gabon, Pacific Costa Rica, Papua New Guinea and Florida (Deem *et al.*, 2006; Harris *et al.*, 2011; Perrault *et al.*, 2012). Additionally, TP concentrations in leatherbacks sampled during the 2009 nesting season were nominally higher than those in leatherbacks sampled from the same beach during the 2006 nesting season (Harris *et al.*, 2011); however, this could be due to methodological differences (Cray and Tatum, 1998; Müller and Brunnberg, 2010). None of the sampled individuals from our study was sampled during the study by Harris *et al.* (2011) of nesting leatherbacks from St Croix (H. Harris, personal communication), so temporal comparisons within individuals across years were not possible.

Nesting turtles are expected to have lower concentrations of TP than foraging turtles because of protein loss during the nesting season (Harris *et al.*, 2011). Nevertheless, Deem *et al.* (2009) found higher TP concentrations in nesting loggerheads (*Caretta caretta*) compared with foraging loggerheads. This was attributed to their reproductive state (i.e., follicle and egg formation trigger protein mobilization). Environmental, spatial, dietary and temporal factors can influence TP concentrations and may explain differences among years, populations and individuals in different physiological states (Osborne *et al.*, 2010).

#### Seasonal trends

Marine turtles rely on the mobilization of fat stores during the nesting season; this is supported by a large decline in plasma triglycerides (Patel, 2013; Plot *et al.*, 2013). Total protein concentrations declined significantly in nesting leatherbacks from St Croix over the nesting season (Fig. 3a), indicating that they are relying on bodily lipids for nutrients. When lipid stores become severely depleted, a shift occurs from fat metabolism to skeletal muscle (i.e., protein) catabolism, which produces the major source of carbon for maintenance of blood glucose levels (Cherel *et al.*, 1988; Hamann *et al.*, 2002; Plot *et al.*, 2013). This shift is evidenced by an increase in blood urea nitrogen, a measure that has value as an indicator of nutritional status. Although we did not measure this metabolite, nesting females from French Guiana exhibited a large dip in blood urea nitrogen at the beginning of the nesting season followed by an increase at the end of the nesting season (Plot *et al.*, 2013). This trend was also observed in Floridian nesting leatherbacks (Perrault *et al.*, 2012), indicating that these animals have entered the protein-catabolizing phase of fasting (Cherel *et al.*, 1988; Plot *et al.*, 2013) and that foraging does not occur while leatherbacks are nesting. However, the increase in blood urea nitrogen could also indicate that these animals have resumed feeding at the end of the nesting season.

Total protein concentrations declined significantly across the nesting season, with the largest decline occurring from the ninth to 10th clutch (mean ± SD = −0.17 ± 0.06 g/dl decline between each nesting event; Fig. 3a). Total protein concentrations during the first clutch averaged 5.7 ± 0.6 g/dl, while concentrations of the eighth, ninth and 10th clutches averaged 4.5 ± 0.5, 4.4 ± 0.4 and 4.1 ± 0.5 g/dl, respectively. Total protein concentrations in nesting leatherbacks from Equatorial Guinea and St Croix were similar during the first (∼5.5 and 5.7 ± 0.2 g/dl, respectively) and fifth nesting events (∼4.5 and 4.9 ± 0.1 g/dl, respectively; Honarvar *et al.*, 2011). This suggests that the decline in TP is similar in nesting leatherbacks across populations. It is unknown if leatherbacks forage during their migrations to nesting grounds (James *et al.*, 2006). Green turtles (*Chelonia mydas*; primarily herbivores) are thought to refrain from foraging during this journey (Hatase *et al.*, 2006). Therefore, TP concentrations could potentially decline during extensive migratory movements (≥4 months; Girondot and Fretey, 1996). However, fasting during feeding-to-breeding ground migration is unlikely in leatherbacks, because foraging individuals from the Atlantic and Pacific Oceans had TP concentrations that were similar (despite methodological differences) to concentrations observed during the early portion of the nesting season at SPNWR (first to fourth nests; Innis *et al.*, 2010; Harris *et al.*, 2011). The results presented here suggest that serum TP concentrations may be used to evaluate whether female turtles are early (first to fourth clutches) or late (fifth clutch or later) in their reproductive cycles during the nesting season.

### Population comparisons

#### Pre-albumin and albumin

In birds and mammals, pre-albumin is composed of the protein transthyretin, which binds the thyroid hormones (Chang *et al.*, 1999). A pre-albumin band was observed in only eight individuals in this study (6% of total samples). In general, pre-albumin is rarely observed or is found at minimal concentrations in marine turtles and other herpetofauna (Harms *et al.*, 1991; Work *et al.*, 2001; Gicking *et al.*, 2004; Deem *et al.*, 2006, 2009). Interestingly, in humans transthyretin is used to indicate malnutrition (Bernstein and Ingenbleek, 2002), and it is plausible that nesting marine turtles have decreased concentrations of this protein due to fasting. This is unlikely, because foraging sea turtles also have a low incidence of this fraction (Gicking *et al.*, 2004; Deem *et al.*, 2009). Pre-albumin has the same mobility as albumin, which may explain why it is masked in agarose gel electrophoresis assays (Lumeij, 2008).

Despite methodological differences, albumin concentrations are similar across all leatherback studies (Table 2), suggesting that albumin concentrations in leatherback turtles are relatively similar within the species and across populations. Albumin concentrations in leatherbacks were higher than the concentrations observed in loggerheads and green turtles of all life stages (Table 2; Gicking *et al.*, 2004; Deem *et al.*, 2009; Osborne *et al.*, 2010). Albumin concentrations in foraging leatherbacks tend to be slightly higher than those of nesting female leatherbacks (Table 2; Deem *et al.*, 2006; Innis *et al.*, 2010; Harris *et al.*, 2011; Honarvar *et al.*, 2011; Perrault *et al.*, 2012; present study), which is likely due to fasting during the nesting season (Honarvar *et al.*, 2011), discussed below (in Seasonal trends of pre-albumin and albumin).

#### Seasonal trends

The albumin fraction is considered to be a negative acute-phase protein, whereby decreases in concentration (relative to normal concentrations) can be related to injury or inflammation (Osborne *et al.*, 2010). Reduced albumin concentrations have been documented in nesting marine turtle studies [hawksbill (*Eretmochelys imbricata*), Goldberg *et al.*, 2013; leatherback, Honarvar *et al.*, 2011; Perrault *et al.*, 2012], most likely resulting from fasting during the nesting season (Zaias and Cray, 2002; Evans, 2011). Additional albumin loss may occur through the production of albumen during amniotic egg formation, because serum albumin is a large component of the egg white (Woodward, 1990). Albumin concentrations in nesting leatherbacks from Equatorial Guinea and St Croix were similar during the first (∼2.0 and 2.1 ± 0.4 g/dl, respectively) and fifth nesting events (∼1.6 and 1.8 ± 0.2 g/dl, respectively; Honarvar *et al.*, 2011). The increase in serum albumin concentrations at the end of the nesting season is puzzling. In nesting hawksbills from Brazil, an appetite-stimulating hormone (ghrelin) was found to increase at the end of the nesting season (Goldberg *et al.*, 2013). If a similar trend occurs in leatherbacks, perhaps nesting females begin to forage opportunistically towards the end of the nesting season, which may explain the increase in serum albumin concentration in the ninth and 10th samples (Fig. 3b; Fossette *et al.*, 2008).

### Acute-phase proteins

#### Population comparisons

Few studies document protein fractions (α-, β- and γ-globulins) in marine turtles (green turtles, Jacobson *et al.*, 2007; Osborne *et al.*, 2010; loggerheads, Gicking *et al.*, 2004; Jacobson *et al.*, 2007; Osborne *et al.*, 2010; leatherbacks, Deem *et al.*, 2006; Innis *et al.*, 2010; Perrault *et al.*, 2012; present study). Concentrations of α_1_-globulins were highest in leatherbacks from St Croix, while concentrations of α_2_-globulins were highest in leatherbacks from Gabon (Deem *et al.*, 2006; Innis *et al.*, 2010). Concentrations of α_1_- and α_2_-globulins were higher in St Croix leatherbacks in comparison to leatherbacks foraging in the western Atlantic (Innis *et al.*, 2010). This finding was intriguing and may reflect inflammation due to injury, because a large number of nesting leatherbacks from St Croix had numerous injuries (from boat strikes, fisheries-related damage, mating injuries or predator attack). Injuries were more prevalent in the Cruzan population in comparison to the Floridian population (J. Perrault, personal observation). Elevated concentrations of α-globulins in nesting females may also be due to physical stress or metabolic status associated with nesting, which may increase bodily concentrations of acute-phase proteins (e.g. α_1_-antitrypsin and α_2_-macroglobulin; Evans, 2011). This trend is similar to that reported by Deem *et al.* (2009), where relatively higher concentrations of α-globulins were found in nesting female loggerheads compared with foraging loggerheads.

Concentrations of the β-globulin fraction were similar across all leatherback studies (Deem *et al.*, 2006; Innis *et al.*, 2010; Perrault *et al.*, 2012; present study); however, β-globulin concentrations in nesting loggerheads from Georgia, USA were higher, by one order of magnitude (on average), than in other marine turtles (Table 2; Deem *et al.*, 2009). β-Globulins consist mainly of acute-phase proteins (e.g. β-lipoprotein; Cray and Tatum, 1998), but some immunoglobulins may be present in this fraction (Osborne *et al.*, 2010; Evans, 2011). In nesting psittacine birds (i.e., parrots), increases in β-globulins have been observed, which may result from an increase in transferrin (Song *et al.*, 1970; Cray and Tatum, 1998), an enzyme that transports iron (Fletcher and Huehns, 1968). Leatherbacks may have a similar mechanism of transferrin-based iron mobilization during nesting; however, transferrin was found to lie in the γ-globulin region in loggerhead turtles (Musquera *et al.*, 1976).

#### Seasonal trends

The α_1_-globulin fraction exhibited a sinusoidal trend during the nesting season (Fig. 3c), while the α_2_-globulin fraction tended to decrease during the nesting season (Fig. 3d). Total α-globulin also tended to decrease, but in a stepwise manner (Fig. 3e). Lastly, the β-globulin fraction decreased across the nesting season; however a slight sinusoidal pattern was observed (Fig. 3f). The α- and β-fractions are positive acute-phase proteins that increase in response to infection and/or inflammation (Walton, 2001), although some immunoglobulins may extend into the β-fraction (Evans, 2011). Concentrations of the acute-phase proteins are expected to decrease in response to fasting and deposition into eggs (Woodward, 1990; Kaneko, 1997; Paltrinieri, 2008), explaining the overall decrease in the α- and β-fractions during the nesting season. Decreases in the α- and β-fractions may also have occurred due to wound healing and the presence of fewer injuries during the end of the nesting season. The acute-phase response (i.e., response to injury or inflammation) exhibits a greater influence on acute-phase protein concentrations than foraging, which may be the reason for the fluctuations of α_1_- and β-globulin fractions during the nesting season (Cerón *et al.*, 2005).

### Immune proteins

#### Population comparisons

The presence of β-γ bridging (Fig. 2c) was found in only six samples (five turtles; 4% of the study samples) in this study. This electrophoretic pattern has also been observed in western Atlantic loggerheads (Gicking *et al.*, 2004), but was not observed in nesting leatherbacks from Gabon, Africa or Florida, USA (Deem *et al.*, 2006; Perrault *et al.*, 2012). The presence of β-γ bridging can indicate chronic disease (including hepatitis) or parasitic infection (Kaneko, 1997; Cray and Tatum, 1998; Gicking *et al.*, 2004). We cannot make inferences about our results in the absence of other plasma biochemical values to assess hepatic disease (e.g. alanine aminotransferase, aspartate aminotransferase and lactate dehydrogenase). Reptiles store fat in the liver (Derickson, 1976), and hepatic stress or liver disease may have occurred as a result of activation of hepatic lipid reserves during vitellogenesis. Perhaps the turtles with β-γ bridging had prior or coincident hepatic disease that was aggravated by egg formation.

In general, concentrations of γ-globulins are higher in foraging marine turtles (Deem *et al.*, 2009; Innis *et al.*, 2010) compared with nesting turtles (Deem *et al.*, 2006, 2009; Perrault *et al.*, 2012; present study). High concentrations of immunoglobulins were observed in wild-caught loggerhead turtles and were attributed to possible trematode infection that stimulated antibody production (Gicking *et al.*, 2004). Immunoglobulin concentrations were lower in juvenile loggerheads compared with adults of the same species (Gicking *et al.*, 2004), which may be attributed to the more mature immune system (due to longer periods of antigenic stimulation) of adults (Innis *et al.*, 2010). Decreased concentrations of γ-globulins can result from immunosuppression (Kaneko, 1997) or differences in antigenic stimulation between foraging and nesting populations (Osborne *et al.*, 2010).

#### Seasonal trends

The γ-globulins tended to decrease in a stepwise manner during the nesting season (Fig. 3g). Long-distance migration may be a large immunostimulant in leatherbacks. These migratory turtles may well encounter a number of antigens during these extensive journeys, which would increase concentrations of γ-globulins (Sibley and Johnsgard, 1959) by the beginning of the nesting season. Normalization of the immunoglobulins may occur during the nesting season while turtle movements are more regional. Leatherbacks experience a decrease in body condition index throughout the nesting season (Plot *et al.*, 2013), further explaining the decrease of immune proteins. Lastly, a number of γ-globulins are deposited into egg albumen, which may also contribute to the stepwise decrease (Woodward, 1990).

During the 2009 nesting season, one of the world's largest oil refineries, HOVENSA, was in operation, producing nearly 500 000 barrels of oil/d (Rae, 2009). Release of toxicants (e.g. heavy metals and polycyclic aromatic hydrocarbons) into local waters where leatherbacks reside during internesting intervals may lead to immune system suppression in these animals (Carlson *et al.*, 2002; Day *et al.*, 2007; Mishra, 2009), with an associated decrease in γ-globulins. Interestingly, blood total mercury concentrations in Cruzan nesting females (Perrault *et al.*, 2013) were negatively correlated with γ-globulins (Spearman rank order correlation; *r*_s_ = −0.24, *P* = 0.01; J. R. Perrault, unpublished data), suggesting that toxicants may be related to a suppression of the immune system in marine turtles.

### Albumin:globulin ratio

#### Population comparisons

Lastly, the A:G ratio is a valuable tool in determining the relative health status of an organism, where lower A:G ratios are indicative of decreased health status (Lumeij, 2008). However, increased A:G ratios do not necessarily reflect better health. The release of glucocorticoids, a class of stress hormones, can suppress globulin production during reproduction (Rafferty and Scheelings, 2014). To support this theory, A:G ratio estimates in nesting leatherbacks were higher than those in foraging leatherbacks (Harris *et al.*, 2011). Additionally, species-specific differences in the A:G ratio are plausible among marine turtles, as is seen in comparisons of bird species (Lumeij, 1987). Nesting and foraging green turtles and leatherbacks had higher A:G ratios than loggerheads (Table 2), possibly as a result of dietary differences. Temporal fluctuations may also be at play (Jacobson *et al.*, 2007). Differences in the A:G ratio are attributable to differences in globulin concentrations. Albumin concentrations were similar across all populations (Table 2), and shifts in globulin may result from antigenic stimulation caused by migrating and nesting (Osborne *et al.*, 2010).

#### Seasonal trends

The A:G ratio was stable throughout the nesting season (Fig. 3i), as a result of a concomitant decrease in albumin and globulin throughout the nesting season (Evans, 2011). We found a slight increase in the A:G ratio towards the end of the nesting season (ninth and 10th samples), which was attributed to an increase in albumins. This slight increase may reflect the resumption of feeding towards the end of the nesting season or follicular atresia (yolk contains a variety of serum proteins; Palmer and Guillette, 1991). We found that albumin tended to decline at a slower rate than globulin (Fig. 4); however, the difference was not enough to affect the A:G ratio, which changed minimally during the nesting season (range, 0.58–0.63 on average). Estrogen induces hyperglobulinaemia during follicle formation (Dessauer, 1970; Campbell, 2006), and we assume that more globulin proteins were mobilized during vitellogenesis, increasing their concentrations at the beginning of the nesting season. This explains the more rapid decrease in globulins relative to albumin at the end of the nesting season.

## Conclusions

Here we provide the following principal findings: (i) the largest sample size of serum proteins and their fractions in nesting leatherbacks; (ii) evidence that some leatherbacks may be immunocompromised during the nesting season; (iii) evidence that leatherback turtles are fasting during the nesting season; and (iv) evidence that reduction in TP may herald the end of a female's nesting allocation for the season. A threshold concentration of TP (∼3.5–4.5 g/dl) may serve as a physiological signal to indicate that the nesting period is complete for individual leatherbacks and that foraging should resume. A critical level of energy depletion is thought to induce the resumption of feeding in marine vertebrates (Groscolas *et al.*, 2008). None of the 76 sampled leatherbacks in this study showed evidence of chronic malnutrition (TP = <3 g/dl; Campbell, 2006), supporting the conclusion that a TP threshold exists. Slight increases in albumin and γ-globulin were observed at the end of the nesting season, suggesting that nesting females are exhibiting follicular atresia (Rostal *et al.*, 1996) and/or beginning to feed before their long-distance migrations back to productive foraging grounds. Significant decreases in some of the protein fractions were not observed, owing to low sample sizes; however, the decreasing trends in the linear regressions (TP, albumin and total globulin) are obvious. The data presented here do not support the hypothesis that nesting leatherbacks from St Croix forage during the nesting season (Eckert *et al.*, 1989) and, instead, confirm the capital breeding hypothesis of Plot *et al.* (2013). Lastly, this study provides insight into the behaviour of leatherback turtles during their internesting intervals, which has proved challenging to population managers and conservationists. We now have a greater understanding of the interactions between leatherback turtle reproduction, fasting and energy reserves during the nesting season, which could improve conservation practices for this internationally endangered species.

## Supplementary material

Supplementary material is available at *Conservation Physiology* online.

Supplementary Data
