# Supplementary material for: Seasonal trends in nesting leatherback turtle (Dermochelys coriacea) serum proteins further verify capital breeding hypothesis
Source: Conserv Physiol. 2014 Feb 18;2(1):cou002. doi: 10.1093/conphys/cou002 (PMC4732470; doi:10.1093/conphys/cou002)
Supplement: Supplementary Data [file supp_2_1_cou002__index.html]

Seasonal trends in nesting leatherback turtle (Dermochelys coriacea) serum proteins further verify capital breeding hypothesis — Supplementary Data 

# Seasonal trends in nesting leatherback turtle (*Dermochelys coriacea*) serum proteins further verify capital breeding hypothesis

## Supplementary Data

Supplementary Data

**Files in this Data Supplement:**

- Supplementary Data - Docx file
