# Supplementary material for: Seasonal trends in nesting leatherback turtle (Dermochelys coriacea) serum proteins further verify capital breeding hypothesis
Source: Conserv Physiol. 2014 Feb 18;2(1):cou002. doi: 10.1093/conphys/cou002 (PMC4732470; doi:10.1093/conphys/cou002)
Supplement: Supplementary Data [file supp_cou002_cou002supp.docx]

| **Supplemental Table 1.** Results of repeated measures ANOVAs for TP, protein fractions and A:G. Numbers in the significant column represent the sample that was significantly higher of the two comparisons. ND represents no difference. | | | | | |
| --- | --- | --- | --- | --- | --- |
|  |  | df | F | *p* | Significant |
| 1 v 2 | TP | 1, 5 | 1.604 | 0.350 | ND |
|  | Albumin | 1, 2 | 14.777 | 0.061 | ND |
|  | Alpha-1 | 1, 2 | 0.116 | 0.766 | ND |
|  | Alpha-2 | 1, 2 | 0.546 | 0.537 | ND |
|  | Alpha | 1, 2 | 0.120 | 0.762 | ND |
|  | Beta | 1, 2 | 1.793 | 0.312 | ND |
|  | Gamma | 1, 2 | 0.419 | 0.584 | ND |
|  | Globulin | 1, 2 | 0.359 | 0.610 | ND |
|  | A:G | 1, 2 | 5.143 | 0.151 | ND |
| 1 v 3 | TP | 1, 6 | 3.195 | 0.124 | ND |
|  | Albumin | 1, 3 | 0.116 | 0.756 | ND |
|  | Alpha-1 | 1, 3 | 0.037 | 0.861 | ND |
|  | Alpha-2 | 1, 3 | 0.558 | 0.509 | ND |
|  | Alpha | 1, 3 | 5.635 | 0.098 | ND |
|  | Beta | 1, 3 | 12.324 | 0.039 | 1 |
|  | Gamma | 1, 3 | 0.024 | 0.886 | ND |
|  | Globulin | 1, 3 | 6.129 | 0.090 | ND |
|  | A:G | 1, 3 | 0.281 | 0.633 | ND |
| 1 v 4 | TP | 1, 6 | 3.877 | 0.096 | ND |
|  | Albumin | 1, 4 | 2.281 | 0.205 | ND |
|  | Alpha-1 | 1, 4 | 0.007 | 0.939 | ND |
|  | Alpha-2 | 1, 4 | 1.334 | 0.312 | ND |
|  | Alpha | 1, 4 | 0.948 | 0.385 | ND |
|  | Beta | 1, 4 | 8.308 | 0.045 | 1 |
|  | Gamma | 1, 4 | 0.054 | 0.828 | ND |
|  | Globulin | 1, 4 | 0.636 | 0.470 | ND |
|  | A:G | 1, 4 | 0.732 | 0.441 | ND |
| 1 v 5 | TP | 1, 3 | 16.667 | 0.027 | 1 |
|  | Albumin | 1, 2 | 1.231 | 0.383 | ND |
|  | Alpha-1 | 1, 2 | 0.951 | 0.432 | ND |
|  | Alpha-2 | 1, 2 | 0.088 | 0.795 | ND |
|  | Alpha | 1, 2 | 50.704 | 0.019 | 1 |
|  | Beta | 1, 2 | 3.020 | 0.224 | ND |
|  | Gamma | 1, 2 | 0.056 | 0.834 | ND |
|  | Globulin | 1, 2 | 25.477 | 0.037 | 1 |
|  | A:G | 1, 2 | 0.420 | 0.583 | ND |
| 1 v 6 | TP | 1, 6 | 27.727 | 0.002 | 1 |
|  | Albumin | 1, 3 | 6.003 | 0.092 | ND |
|  | Alpha-1 | 1, 3 | 0.175 | 0.704 | ND |
|  | Alpha-2 | 1, 3 | 0.359 | 0.591 | ND |
|  | Alpha | 1, 3 | 0.161 | 0.715 | ND |
|  | Beta | 1, 3 | 5.350 | 0.104 | ND |
|  | Gamma | 1, 3 | 0.030 | 0.873 | ND |
|  | Globulin | 1, 3 | 8.829 | 0.059 | ND |
|  | A:G | 1, 3 | 0.022 | 0.891 | ND |
| 1 v 7 | TP | 1, 3 | 10.862 | 0.046 | 1 |
|  | Albumin | 1, 2 | 2.249 | 0.272 | ND |
|  | Alpha-1 | 1, 2 | 2.973 | 0.227 | ND |
|  | Alpha-2 | 1, 2 | 0.680 | 0.496 | ND |
|  | Alpha | 1, 2 | 2.975 | 0.227 | ND |
|  | Beta | 1, 2 | 5.411 | 0.146 | ND |
|  | Gamma | 1, 2 | 0.619 | 0.514 | ND |
|  | Globulin | 1, 2 | 7.654 | 0.110 | ND |
|  | A:G | 1, 2 | 5.323 | 0.147 | ND |
| 2 v 3 | TP | 1, 11 | 0.200 | 0.663 | ND |
|  | Albumin | 1, 5 | 0.196 | 0.676 | ND |
|  | Alpha-1 | 1, 5 | 4.812 | 0.080 | ND |
|  | Alpha-2 | 1, 5 | 1.826 | 0.234 | ND |
|  | Alpha | 1, 5 | 5.617 | 0.064 | ND |
|  | Beta | 1, 5 | 2.123 | 0.205 | ND |
|  | Gamma | 1, 5 | 2.538 | 0.172 | ND |
|  | Globulin | 1, 5 | 7.636 | 0.040 | 2 |
|  | A:G | 1, 5 | 2.388 | 0.183 | ND |
| 2 v 4 | TP | 1, 9 | 16.644 | 0.003 | 2 |
|  | Albumin | 1, 5 | 24.054 | 0.004 | 2 |
|  | Alpha-1 | 1, 5 | 0.067 | 0.806 | ND |
|  | Alpha-2 | 1, 5 | 5.272 | 0.070 | ND |
|  | Alpha | 1, 5 | 5.170 | 0.072 | ND |
|  | Beta | 1, 5 | 0.831 | 0.404 | ND |
|  | Gamma | 1, 5 | 0.717 | 0.436 | ND |
|  | Globulin | 1, 5 | 7.797 | 0.038 | 2 |
|  | A:G | 1, 5 | 0.910 | 0.384 | ND |
| 2 v 5 | TP | 1, 8 | 5.108 | 0.054 | 2 |
|  | Albumin | 1, 6 | 10.447 | 0.018 | 2 |
|  | Alpha-1 | 1, 6 | 0.024 | 0.883 | ND |
|  | Alpha-2 | 1, 6 | 3.443 | 0.113 | ND |
|  | Alpha | 1, 6 | 11.835 | 0.014 | 2 |
|  | Beta | 1, 6 | 0.656 | 0.449 | ND |
|  | Gamma | 1, 6 | 0.556 | 0.484 | ND |
|  | Globulin | 1, 6 | 1.042 | 0.347 | ND |
|  | A:G | 1, 6 | 1.507 | 0.266 | ND |
| 2 v 6 | TP | 1, 9 | 10.770 | 0.010 | 2 |
|  | Albumin | 1, 4 | 11.619 | 0.027 | 2 |
|  | Alpha-1 | 1, 4 | 0.107 | 0.760 | ND |
|  | Alpha-2 | 1, 4 | 0.970 | 0.380 | ND |
|  | Alpha | 1, 4 | 0.765 | 0.431 | ND |
|  | Beta | 1, 4 | 0.266 | 0.633 | ND |
|  | Gamma | 1, 4 | 0.179 | 0.694 | ND |
|  | Globulin | 1, 4 | 0.354 | 0.584 | ND |
|  | A:G | 1, 4 | 8.659 | 0.042 | 2 |
| 2 v 7 | TP | 1, 5 | 26.786 | 0.004 | 2 |
|  | Albumin | 1, 3 | 29.208 | 0.012 | 2 |
|  | Alpha-1 | 1, 3 | 0.316 | 0.613 | ND |
|  | Alpha-2 | 1, 3 | 2.625 | 0.204 | ND |
|  | Alpha | 1, 3 | 9.022 | 0.058 | ND |
|  | Beta | 1, 3 | 0.168 | 0.710 | ND |
|  | Gamma | 1, 3 | 0.689 | 0.467 | ND |
|  | Globulin | 1, 3 | 2.910 | 0.187 | ND |
|  | A:G | 1, 3 | 0.073 | 0.805 | ND |
| 2 v 8 | TP | 1, 8 | 106.480 | <0.001 | 2 |
|  | Albumin | 1, 6 | 275.534 | <0.001 | 2 |
|  | Alpha-1 | 1, 6 | 0.620 | 0.461 | ND |
|  | Alpha-2 | 1, 6 | 3.165 | 0.118 | ND |
|  | Alpha | 1, 6 | 3.241 | 0.122 | ND |
|  | Beta | 1, 6 | 1.646 | 0.247 | ND |
|  | Gamma | 1, 6 | 0.380 | 0.560 | ND |
|  | Globulin | 1, 6 | 21.450 | 0.004 | 2 |
|  | A:G | 1, 6 | 3.689 | 0.103 | ND |
| 2 v 9 | TP | 1, 3 | 70.810 | 0.004 | 2 |
| 2 v 10 | TP | 1, 2 | 600.250 | 0.002 | 2 |
| 3 v 4 | TP | 1, 11 | 15.751 | 0.002 | 3 |
|  | Albumin | 1, 7 | 7.344 | 0.030 | 3 |
|  | Alpha-1 | 1, 7 | 1.428 | 0.271 | ND |
|  | Alpha-2 | 1, 7 | 0.394 | 0.550 | ND |
|  | Alpha | 1, 7 | 0.170 | 0.692 | ND |
|  | Beta | 1, 7 | 0.966 | 0.358 | ND |
|  | Gamma | 1, 7 | 0.106 | 0.755 | ND |
|  | Globulin | 1, 7 | 0.204 | 0.665 | ND |
|  | A:G | 1, 7 | 2.842 | 0.136 | ND |
| 3 v 5 | TP | 1, 12 | 46.085 | <0.001 | 3 |
|  | Albumin | 1, 7 | 46.872 | <0.001 | 3 |
|  | Alpha-1 | 1, 7 | 1.424 | 0.272 | ND |
|  | Alpha-2 | 1, 7 | 2.422 | 0.164 | ND |
|  | Alpha | 1, 7 | 1.102 | 0.329 | ND |
|  | Beta | 1, 7 | 0.690 | 0.433 | ND |
|  | Gamma | 1, 7 | 0.208 | 0.665 | ND |
|  | Globulin | 1, 7 | 7.923 | 0.026 | 3 |
|  | A:G | 1, 7 | 0.093 | 0.769 | ND |
| 3 v 6 | TP | 1, 11 | 54.969 | <0.001 | 3 |
|  | Albumin | 1, 5 | 21.746 | 0.006 | 3 |
|  | Alpha-1 | 1, 5 | 0.002 | 0.968 | ND |
|  | Alpha-2 | 1, 5 | 5.021 | 0.075 | ND |
|  | Alpha | 1, 5 | 2.402 | 0.182 | ND |
|  | Beta | 1, 5 | 1.185 | 0.326 | ND |
|  | Gamma | 1, 5 | 0.478 | 0.520 | ND |
|  | Globulin | 1, 5 | 18.429 | 0.008 | 3 |
|  | A:G | 1, 5 | 0.093 | 0.773 | ND |
| 3 v 7 | TP | 1, 4 | 23.972 | 0.008 | 3 |
| 3 v 8 | TP | 1, 5 | 93.913 | <0.001 | 3 |
| 3 v 9 | TP | 1, 2 | 270.750 | 0.004 | 3 |
| 4 v 5 | TP | 1, 14 | 1.485 | 0.243 | ND |
|  | Albumin | 1, 11 | 1.186 | 0.299 | ND |
|  | Alpha-1 | 1, 11 | 0.160 | 0.696 | ND |
|  | Alpha-2 | 1, 11 | 1.127 | 0.311 | ND |
|  | Alpha | 1, 11 | 1.348 | 0.270 | ND |
|  | Beta | 1, 11 | 0.052 | 0.824 | ND |
|  | Gamma | 1, 11 | 0.147 | 0.709 | ND |
|  | Globulin | 1, 11 | 1.014 | 0.336 | ND |
|  | A:G | 1, 11 | 0.204 | 0.660 | ND |
| 4 v 6 | TP | 1,14 | 4.183 | 0.060 | ND |
|  | Albumin | 1, 7 | 1.547 | 0.254 | ND |
|  | Alpha-1 | 1, 7 | 0.473 | 0.514 | ND |
|  | Alpha-2 | 1, 7 | 0.087 | 0.776 | ND |
|  | Alpha | 1, 7 | 0.738 | 0.419 | ND |
|  | Beta | 1, 7 | 0.328 | 0.585 | ND |
|  | Gamma | 1, 7 | 0.038 | 0.850 | ND |
|  | Globulin | 1, 7 | 0.047 | 0.835 | ND |
|  | A:G | 1, 7 | 0.033 | 0.860 | ND |
| 4 v 7 | TP | 1, 8 | 40.042 | <0.001 | 4 |
|  | Albumin | 1, 3 | 8.234 | 0.064 | ND |
|  | Alpha-1 | 1, 3 | 5.411 | 0.103 | ND |
|  | Alpha-2 | 1, 3 | 0.650 | 0.479 | ND |
|  | Alpha | 1, 3 | 12.075 | 0.040 | 4 |
|  | Beta | 1, 3 | 0.290 | 0.628 | ND |
|  | Gamma | 1, 3 | 2.848 | 0.190 | ND |
|  | Globulin | 1, 3 | 10.236 | 0.049 | 4 |
|  | A:G | 1, 3 | 0.719 | 0.459 | ND |
| 4 v 8 | TP | 1, 5 | 49.706 | 0.001 | 4 |
| 4 v 9 | TP | 1, 3 | 78.000 | 0.003 | 4 |
| 4 v 10 | TP | 1, 2 | 142.231 | 0.007 | 4 |
| 5 v 6 | TP | 1, 17 | 8.532 | 0.010 | 5 |
|  | Albumin | 1, 11 | 18.519 | 0.001 | 5 |
|  | Alpha-1 | 1, 11 | 0.375 | 0.553 | ND |
|  | Alpha-2 | 1, 11 | 0.033 | 0.860 | ND |
|  | Alpha | 1, 11 | 0.352 | 0.565 | ND |
|  | Beta | 1, 11 | 1.915 | 0.194 | ND |
|  | Gamma | 1, 11 | 2.097 | 0.175 | ND |
|  | Globulin | 1, 11 | 4.590 | 0.055 | ND |
|  | A:G | 1, 11 | 0.353 | 0.564 | ND |
| 5 v 7 | TP | 1, 9 | 5.287 | 0.047 | 5 |
|  | Albumin | 1, 5 | 24.951 | 0.004 | 5 |
|  | Alpha-1 | 1, 5 | 1.448 | 0.283 | ND |
|  | Alpha-2 | 1, 5 | 0.311 | 0.616 | ND |
|  | Alpha | 1, 5 | 0.260 | 0.632 | ND |
|  | Beta | 1, 5 | 0.001 | 0.980 | ND |
|  | Gamma | 1, 5 | 3.642 | 0.129 | ND |
|  | Globulin | 1, 5 | 2.907 | 0.149 | ND |
|  | A:G | 1, 5 | 0.006 | 0.941 | ND |
| 5 v 8 | TP | 1, 5 | 98.455 | <0.001 | 5 |
|  | Albumin | 1, 3 | 13.353 | 0.030 | 5 |
|  | Alpha-1 | 1, 3 | 5.049 | 0.154 | ND |
|  | Alpha-2 | 1, 3 | 1.469 | 0.312 | ND |
|  | Alpha | 1, 3 | 4.846 | 0.115 | ND |
|  | Beta | 1, 3 | 0.994 | 0.392 | ND |
|  | Gamma | 1, 3 | 0.183 | 0.697 | ND |
|  | Globulin | 1, 3 | 19.158 | 0.022 | 5 |
|  | A:G | 1, 3 | 0.159 | 0.717 | ND |
| 5 v 9 | TP | 1, 3 | 86.783 | 0.003 | 5 |
| 5 v 10 | TP | 1, 2 | 45.432 | 0.021 | 5 |
| 6 v 7 | TP | 1, 11 | 0.041 | 0.843 | ND |
|  | Albumin | 1, 5 | 2.000 | 0.216 | ND |
|  | Alpha-1 | 1, 5 | 0.097 | 0.768 | ND |
|  | Alpha-2 | 1, 5 | 1.227 | 0.318 | ND |
|  | Alpha | 1, 5 | 2.325 | 0.188 | ND |
|  | Beta | 1, 5 | 0.723 | 0.434 | ND |
|  | Gamma | 1, 5 | 1.138 | 0.346 | ND |
|  | Globulin | 1, 5 | 0.026 | 0.878 | ND |
|  | A:G | 1, 5 | 0.577 | 0.482 | ND |
| 6 v 8 | TP | 1, 5 | 20.082 | 0.007 | 6 |
|  | Albumin | 1, 3 | 31.579 | 0.011 | 6 |
|  | Alpha-1 | 1, 3 | 0.010 | 0.927 | ND |
|  | Alpha-2 | 1, 3 | 0.834 | 0.428 | ND |
|  | Alpha | 1, 3 | 1.470 | 0.312 | ND |
|  | Beta | 1, 3 | 2.106 | 0.243 | ND |
|  | Gamma | 1, 3 | 4.800 | 0.116 | ND |
|  | Globulin | 1, 3 | 40.961 | 0.008 | 6 |
|  | A:G | 1, 3 | 0.026 | 0.886 | ND |
| 6 v 9 | TP | 1, 2 | 137.286 | 0.007 | 6 |
| 7 v 8 | TP | 1, 5 | 20.851 | 0.006 | 7 |
|  | Albumin | 1, 3 | 5.681 | 0.097 | ND |
|  | Alpha-1 | 1, 3 | 20.535 | 0.020 | 7 |
|  | Alpha-2 | 1, 3 | 0.544 | 0.514 | ND |
|  | Alpha | 1, 3 | 2.591 | 0.206 | ND |
|  | Beta | 1, 3 | 0.478 | 0.539 | ND |
|  | Gamma | 1, 3 | 0.267 | 0.641 | ND |
|  | Globulin | 1, 3 | 4.751 | 0.117 | ND |
|  | A:G | 1, 3 | 0.207 | 0.680 | ND |
| 7 v 9 | TP | 1, 2 | 9.757 | 0.089 | ND |
| 8 v 9 | TP | 1, 4 | 5.063 | 0.088 | ND |
| 8 v 10 | TP | 1, 3 | 16.667 | 0.027 | 8 |
| 9 v 10 | TP | 1, 4 | 5.365 | 0.081 | ND |
|  | Albumin | 1, 3 | 1.434 | 0.317 | ND |
|  | Alpha-1 | 1, 3 | 2.407 | 0.219 | ND |
|  | Alpha-2 | 1, 3 | 2.983 | 0.183 | ND |
|  | Alpha | 1, 3 | 9.232 | 0.056 | ND |
|  | Beta | 1, 3 | 3.051 | 0.179 | ND |
|  | Gamma | 1, 3 | 40.333 | 0.008 | 10 |
|  | Globulin | 1, 3 | 6.142 | 0.089 | ND |
|  | A:G | 1, 3 | 0.558 | 0.509 | ND |
